# In vitro characterization the antioxidant and antibacterial properties of hemp (*Cannabis sativa* spp.) varieties cultivated in Northern Alabama

**DOI:** 10.1186/s42238-025-00258-y

**Published:** 2025-01-31

**Authors:** Aaron Dudley, Lamin S. Kassama, Armitra Jackson-Davis, Ernst Cebert, Xianyan Kuang

**Affiliations:** 1 Department of Food and Animal Sciences, Alabama A&M University, A-101 Carver Complex Thomas Wing, 4900 Meridian St, Normal, AL 35762 USA; 2 Department of Natural Resources & Environmental Sciences, Alabama A&M University, 4900 Meridian St, Normal, AL 35762 USA

**Keywords:** Antimicrobial, Antioxidant, BioScreen C, Growth inhibition, Northern alabama hemp, Radical scavenging activity

## Abstract

Plants, especially those recognized for their medicinal properties, are an excellent source of bioactive components and are attracting considerable interest in the food industry due to their natural bioactivity. In this context, hemp species (*Cannabis sativa* spp.) were investigated for such applications because of their well-documented antibacterial and antioxidant activities. However, the bioactive efficacy of varieties being introduced in Northern Alabama and their implications for food safety have not been studied. The purpose of this study was to evaluate the antibacterial and antioxidative potential of four hemp varieties grown at the Alabama A&M University, Winfred Thomas Agricultural Research Station in Northern Alabama using three different extraction solvents (deionized water, acetone, and ethanol). Antioxidant potential was evaluated by DPPH free radical scavenging activity (2, 2-diphenyl-1- picrylhydrazyl), Total phenolic and flavonoid contents. Antibacterial activity against cocktails of enteric pathogens, including *Listeria monocytogenese*, *E. coli* O157:H7, and *Salmonella enterica* was evaluated for optical density using a BioScreen-C microtiter. Also, the disc diffusion extraction yield was evaluated to determine the best extraction solvent. Data were expressed as mean ± standard error (*n* = 3) and ANOVA (*P* ≤ 0.05). The ethanolic extracts exhibited the the highest extraction yield at 25.29 ± 0.70% (RE), while the antioxidant result demonstrated that the ethanolic extracts had the highest DPPH free radical scavenging activity at 64.03 ± 0.26% (RE). The results of the antibacterial studies showed that ethanolic hemp extracts exhibited significantly higher growth inhibition against all foodborne pathogens > 70% (*p* ≤ 0.05). The results show that the ethanolic extracts has significant extraction yield and bioactivity, highlighting ethanolic extract utilization in future antimicrobial nanofiber application.

## Introduction

The global population is projected to grow exponentially from 7.5 billion to 8.5 billion within the next decade (UN, 2015). Hence, reducing food spoilage and preventing foodborne outbreaks will be critical in sustaining the food supply chain and providing safe food to the growing population. The CDC estimates that every year, 17% of Americans become ill, hospitalized, or die from foodborne illnesses (Scallan et al. 2011). Thirty-one significant pathogens cause foodborne disease, with the top five most common are: *Listeria monocytogenes*, *Salmonella enterica*, *Campylobacter jejuni*, *Escherichia coli 0157:H7*, and *Clostridium perfringens*.

Mitigating the prevalence of these pathogens, chemical preservatives are used by food manufacturers to decontaminate food contact surfaces. These come in the form of halogens (sodium hypochlorite and chlorine oxide), organic acids (acetic, lactic, citric acids), and metal ions (Ag^+^, Zn^+^, Cu^2+^). These synthetic additives are under public scrutiny due to consumers’ perceived toxicity and negative organoleptic attributes. Apparently, to meet the consumer requirements, manufacturers are seeking natural alternatives derived from plant sources (Yuan and Yuk 2018). Bioactive plants are viable alternatives to the synthetic decontaminants because of their antibacterial and antioxidative activities associated with their polyphenolic contents. One such plant-based bioactive material gaining interest within the food industry is Hemp (*Cannabis sativa*). *Cannabis sativa* has been used for thousands of years to prevent disease in humans. Hemp aerial plant parts (leaves, flowers, and stem) are rich in secondary plant metabolites of polyphenolic groups: terpenes, phenolics (flavonoids and phenolic acids), and terphenophenolics (cannabinoids).

These polyphenolic compounds are obtained in numerous ways, including drying and milling or grinding parts of plants, but the most common method is solvent extraction (Putri et al., 2019). Extraction is the primary method used to recover and isolate polyphenolic compounds from plants. The most widely used and cost-effective process is maceration. Maceration is a solid-liquid extraction process by which a ground plant material is dispersed into a liquid medium to recover compounds with chemical affinity similar to that of the solvent. The factors that influence the maceration process include particle size, solvent polarity, pH extraction temperature, extraction time, and solid-to-liquid ratio (Drinic et al., 2018; Putri et al., 2019). Manipulation of the solvent is one of the most effective strategies for maceration, and the common solvents used for hemp plants are ethanol, methanol, water, acetone, and/or their combinations. Hence, a significant challenge is the optimization of the extraction parameters to obtain the best yield with potent antioxidant and antimicrobial activity.

Research has demonstrated that *Cannabis* has antibacterial properties against bacteria (Ahmed et al., 2019; Chakraborty et al., 2018; Anjum et al. 2018; Anumudu et al. 2020; Das and Mishra, 2011; Frassinetti et al. 2020; Nissen et al. 2010; Muscara et al., 2020). Das and Mishra (2011) evaluated the zone of inhibition technique using petroleum ether extracts from *Cannabis* leaf on *E. coli* ATCC 10536 and found antibacterial effectiveness by inhibiting bacterial growth. Muscara et al. (2020) evaluated the Minimum Inhibitory Concentration (MIC) assay of Futura, a low tetrahydrocannabinol (THC) Hemp variety against gram-positive and gram-negative bacteria. They found that Futura essential oils significantly inhibited microbial growth, and thus concluded that the Futura variety has great potential to inhibit foodborne pathogens and minimize food spoilage. The antioxidative bioactivity of *Cannabis sativa* is attributed to the presence of phenolic and terphenophenolic groups that scavenge free radicals and prevent oxidative stress, which can lead to inflammation, cancer, and reduced shelf life and consumer satisfaction due to changes in the appearance of foods (Drinic et al., 2018). Several studies have reported the effectiveness of *Cannabis* extracts in preventing free radical propagation (Drinic et al., 2018; Irakli et al. 2019; Muscara et al., 2020).

Bioactive compounds derived from *Cannabis sativa* plants have been well studied. However, due to international narcotic regulations, its benefits as a food preservative are yet to be studied. The 2014 and 2018 Farm Bill passages in the United States Congress removed hemp from the Schedule-I narcotics list. In the US, institutions of higher education and state agricultural departments can grow hemp varieties that contain not greater than 0.3% (w/w) of the psychoactive cannabinoid delta-9-THC in dried plant material (Rupasinghe et al., 2020; FDA, 2023). Although hemp has known antimicrobial and antioxidative properties, little is known about the bioactivity of Northern Alabama hemp varieties and their implications in food safety applications. Therefore, the purpose of present study was to evaluate the four varieties of Northern Alabama Grown hemp extracts in three different extraction solvents, and to optimize the extraction yield, antibacterial, and antioxidant properties.

## Materials and methods

### Chemicals

The following solvents: 94–96% ethanol, acetone, and granular sodium nitrite, were obtained from Alfa Aesar (Ward Hill, Ma). DPPH (2,2 Diphenyl-1-2-picryl-hydrazyl) was obtained from MedChem Express (Monmouth Junction, NJ). Anhydrous aluminum chloride and sodium carbonate were purchased from Fisher Chemical (Ottawa, ON). 1.0 M Sodium Hydroxide and Quercetin (Sigma-Aldrich, St. Louis, Mo), Anhydrous Gallic Acid (Acros Organics, Geel, Belgium), and Folin-Ciocalteu Phenol reagent (Spectrum chemical, New Brunswick, NJ) were purchased for the total phenolic content, total flavonoid content, and DPPH assays. All chemicals had a purity of analytical grade or better.

### Bacterial media

Bacterial media preparation was adapted from the methods of Frassinetti et al. (2020) using Tryptone soy (TSB) and tryptone soy yeast extract (TSBYE) broth (Oxoid, Cheshire, England) were prepared as follows: 30 g of broth powder was dissolved in 1000 mL of deionized water and sterilized by autoclaving at 121 °C for 15 min. Broth compositions for TSB and TSBYE were as follows: Pancreatic digest of casein 17.0 g/L, enzymatic digest of soybean 3.0 g/L, sodium chloride 5.0 g/L, dipotassium hydrogen phosphate 2.5 g/L, and glucose 2.5 g/L; and pancreatic digest of casein 17.0 g/L, dextrose 2.5 g/L, dipotassium hydrogen phosphate 2.5 g/L, the enzymatic digest of soybean 3.0 g/L, sodium chloride 5.0 g/L, and yeast extract 6.0 g/L with a final pH of 7.3 ± 0.2 (25 °C), respectively.

### Bacterial strain and culture media

The antibacterial activity of *Cannabis* extracts was evaluated against three bacterial strains known to cause foodborne pathogenesis. Pathogenic bacterial strains were acquired from the Food Microbiology Laboratory, Iowa State University (Ames, IA) and the United States Department of Agriculture, Food Safety and Inspection Service Food Microbiology Laboratory (Athens, Ga). Microbiological analysis was performed using gram-positive strains of *Listeria monocytogenes* (LM) (H7969, H7962, and Scott A NADC 2045 serovar 4b), gram-negative serovars of *Escherichia coli* 0157:H7 (ATCC 35150, ATCC 43894, and FRIK 125), and serovars (SE) of *Salmonella enterica* spp. (FSIS32105652-serovar *Typhimurium*, FSIS32105654-serovar *Enteritidis*, and FSIS32105656-serovar *Infantis*).

Serovars were blended into a cocktail by combining 1mL of each strain into a 15mL test tube and filling that test tube with 12mL of TSB or TSBYE. Serovars were blended into a cocktail by adding 1 mL of each strain into a 15 mL test tube, which was then filled with 12 mL of TSB or TSBYE. Thereafter, the cocktails were isolated onto Sorbitol MacConkey agar (Oxoid, Cheshire, England) for *E. coli* O157:H7, Modified Oxford agar (Oxoid, Cheshire, England) for *Listeria monocytogenes*, and Xylose-Lysine-Deoxycholate (Oxoid, Cheshire, England) for *Salmonella enterica* spp. Then the strains were enumerated, isolated in respective broths, and kept in an incubator at 37 °C prior to analysis.

### Sample preparation

*Cannabis sativa* L varieties were obtained from the Alabama A&M Winfred Thomas Agricultural Research Station (N 34.9025-W 86.5596) in Hazel Green, Alabama. The varieties were chosen because of their successful growth in the northern Alabama climate. The following hemp varieties that were used in this study were: Jinma 1 (J1), Cherry Wine (CW), Rogue (R), and Queen Dream (QD). The hemp samples were collected directly from the field at the end of the growing season. Hemp female inflorescences, which were certified for a content of delta-9-THC below 0.3% (w/w), of the *Cannabis sativa* L plant were harvested from the field and transported to the AAMU research pilot plant for drying. The drying process was conducted using a cabinet dryer (Proctor and Schwartz Inc., Horsham, PA) set at 46 °C for 24 h. After drying, the inflorescences were ground into a fine powder with particle sizes ranging from 250 to 800 μm using a coffee grinder (Black and Decker Smart Grind, Towson, MD). The ground samples were sealed in a zip-lock bag and stored at ambient temperature (25 °C) until use.

### Extraction of bioactives

*Cannabis sativa* L inflorescence extraction was performed as described by Khanal et al. (2009) with modifications. Ground plant inflorescence (2 g) was suspended in a 50 mL centrifuge tube with 15mL of extraction solvent (ethanol, acetone, and deionized water) and agitated and incubated for 24 h at 25 °C.After incubation, the extract was centrifuged (Thermo Scientific, Sorvall ST 40 Centrifuge, Waltham, MA) at 2500 g for 8 m. The supernatant was collected, filtered with Whatman filter paper (#4, 4.7 cm diameter, 25 μm pore size, Piscataway, NJ), kept in a 50 mL centrifuge tube wrapped in aluminum foil, and stored at 4 °C until use.

### Quantitative physiochemical analysis

#### Determination of extraction yield

The extraction yield was determined by the modified method developed by Oroian et al. (2020). The remaining residue from the Hemp extraction was weighed and dried in a convection oven (Quincy Lab Inc, Model 30 GC Lab Oven, Chicago, IL) for 20 h at 60 °C. The extraction yield was calculated (Eq. 1) as follows:

(%) Extraction Yield = ($$\:\frac{{M}_{1}-{M}_{2}}{{M}_{1}}$$) × 100 (1)

where M_1_ is the weight of dried ground Hemp sample before extraction, and M_2_ is the weight of the Hemp extraction residue after drying.

#### Determination of total phenolic content (TPC)

The TPC of the aliquots was determined to assess the phytochemical content and antioxidant activities, following the method described by Gajula et al. (2009) using the Folin-Ciocalteu reagent. Gallic acid standard concentrations of 0.02, 0.04, 0.06, 0.08, and 0.10 mg/mL were prepared. The sample and standard solutions (12.5 µL) were pipetted into the 96-well plate cells. Folin-Ciocalteu solution (12.5 µL) was added to each well and incubated for 5 min. Then, 125 µL of 7% sodium carbonate was added, followed by 50 µL of deionized water. The absorbance of the mixture was measured at 750 nm using a microplate spectrophotometer (SpectraMax 150, Molecular Devices, Sunnyvale, CA). The results were expressed as milligrams of gallic acid equivalents (GAE) per gram of the dry weight of hemp powder (mg GAE/g DW).

### Determination of total flavonoids content (TFC)

The TFC of the aliquots was determined to assess the phytochemical and antioxidant activities using the method described by Gajula et al. (2009). The standard solutions of catechin concentrations prepared were 0.02, 0.04, 0.06, 0.08, and 0.10 mg/mL. The samples and/or the standard solutions (25 µL) were added to the 96-well plate cells, and 7.5 µL of sodium nitrite was added, followed by an incubation period of 5 min. This was followed by adding and incubating 15 µL of aluminum chloride for 5 min, then 50 µL of sodium hydroxide was added and left for 5 min, and finally, 40 µL of deionized water was subsequently added. Hence, the absorbance was read at 510 nm using a microplate spectrophotometer (SpectraMax 150, Molecular Devices, Sunnyvale, California). The TFC quantity was determined using the catechin acid standard curve, and the results were expressed as milligrams of Quercetin equivalents (QE) per gram of the dry weight of hemp powder (mg QE/gram DW).

### Determination of 2, 2-diphenyl-2-picrylhydrazyl (DPPH) scavenging capacity

The aliquots’ DPPH radical scavenging assay was conducted using the modified method of Chen et al. (2014). DPPH stock solutions were prepared daily with 0.001 g of DPPH and 50 mL of 80% ethanol before measurement using the microplate spectrophotometer (SpectraMax 150, Molecular Devices, Sunnyvale, California). The standard or the sample (40 µL helm extract) at various concentrations was placed in a 96-well microplate. Subsequently, 200 µL of freshly prepared DPPH stock solution was added. The absorbance was measured at 517 nm against a blank at 30 min intervals for 90 min using a microplate spectrophotometer (SpectraMax 150, Molecular Devices, Sunnyvale, California). Each sample was measured in triplicate (*n* = 3). The DPPH scavenging effect was calculated using Eq. 2:

(%) Inhibition = ($$\:\frac{Acontrol-{Asample}_{}}{Acontrol}$$) × 100 (2)

where A_ontrol_ is the the absorbance of the blank and A_sample_ is the absorbance of the sample extract.

### Microbiological analysis

#### Descriptive microbial growth profile of antimicrobial

The descriptive microbial growth profile was obtained using the BioScreen C Microbiological Growth Analyzer (Lab systems, Helsinki, Finland). The method was adapted from Media et al. (2012) and modified. A 96-well microtiter plate was loaded with 50 µL pathogen (*Listeria monocytogenes*,* E. coli* O157:H7, *and Salmonella enterica* cocktail ~ 10^6^ CFU/mL) and 150 µL hemp extract. The optical density (OD) was measured at 600 nm and recorded every 30 min for 24 h at 37 °C. The data was recorded using the EZ Experiment software provided by the manufacturer. The data was then exported to Microsoft Excel (Microsoft Corporation, Redmond, WA, USA) for further analysis. The percentage of microbial inhibition was calculated using Eq. 3:

(%) Microbial Growth Inhibition = ($\:\frac{Acontrol-{Asample}_{}}{Acontrol}$) × 100 (3)

where A_ontrol_ is the absorbance of the inoculated broth, and A_sample_ is the absorbance of the hemp extract/inoculated broth.

### Disc diffusion assay

The plant extracts were sterilized using a 30 cc syringe with a self-locking double-layer 0.8 μm pore size prior to the study. The disc diffusion method was adapted from Hudzicki (2009), Mkpenie et al. (2012), and Rubab et al. (2021) with modifications. The cell suspension was prepared by loop inoculation of an 18-h bacterial culture cocktail in 5 mL of 0.85% sterile saline solution. The turbidity of the cell suspension was matched with 0.5 McFarland standards, equivalent to a microbial load of 1.5 × 10^8^ CFU/mL. A microbial load was inoculated onto Mueller Hinton Agar (MHA) plates (Oxoid, UK) and allowed to air dry at room temperature (~ 25 °C) for 10 min.

Disc diffusion method was used toimpregnate 6 mm sterile cotton discs (Carolina Biological Supply, Burlington, MA) with 100 µL of different Hemp extract treatments. These discs were then placed on Mueller-Hinton Agar (MHA) and allowed to diffuse into the media for five minutes. Positive controls (ethanol, acetone, and distilled deionized water) and negative controls were also included. Each treatment was placed on individual plates. The MHA plates were then placed into an incubator (Heratherm IG S60, Thermo Scientific, Waltham, MA) set at 37 °C for 24 h. After incubation, the antibacterial activity of each disc was determined by measuring the diameter (mm) of the zones using a caliper. Therefore, zones of inhibition < 8 mm are considered to be resistant.

### Statistical analysis

A 4 × 3 full factorial design was used with two factors: Hemp variety and extraction solvent. There were four levels of Hemp Variety (Jinma 1, Cherry Wine (CW), Queen Dream (QD), Rogue (R)) and three levels of extraction solvents (Ethanol (E), Acetone (AE), and Deionized Distilled Water (DIW)) utilized. Data were statistically analyzed using SAS 9.4 software (SAS Institute, Cary, NC). Hence, the analysis of variance (ANOVA) and Tukey’s post hoc comparison test were used to analyze further the experimental treatments that were found to be significant (*p* < 0.05). All the experiments were conducted in triplicate (*n* = 3). Pearson correlation and Principal Component Analysis (PCA) were used on the data to determine the relationships and optimize the effects of various parameters.

## Results

### Extraction yield

Extraction yield (EY) was evaluated in this experiment and was found to vary in the range of 4.79 ± 0.02 for J1 DIW and 25.29 ± 0.70% for RE (as shown in Table 1). Table 2 shows the concentrations of the extracts based on the hemp variety and extraction solvent. The extraction yield is dependent on the solubility of the phytochemical compounds in the extraction solvent. The key extraction parameters, such as solid-liquid ratio, particle size, and extraction time, were kept constant, in line with the study of Suleria et al. (2020). The components in the ground hemp dissolved in the solvent with similar polarity, indicating that the EY is linked to the solvent that solubilizes the most phytochemicals (Putri et al., 2019). There were significant differences (*p* ≤ 0.05) between the solvent types and hemp varieties used in the experiment. Consequently, the ethanol and acetone extracts had significantly higher EY (*p* < 0.05) than the DIW extracts. The experimental results also show that the hemp variety significantly influenced the EY. The QD variety exhibited the highest EY, while the Jinma 1 showed the lowest. The differences could be attributed to biotic and abiotic factors, such as climate, elevation, rainfall, temperature, genetic factors, soil characteristics, and duration and intensity of light exposure, which impact the chemotype (chemical phenotype, that is, phytochemical composition and profile), shikimic and phenyl propanoic acids contents. Thus, causing variations in the phytochemicals produced in different varieties of hemp plants grown at different geographical locations (Giupponi et al. 2020).

**Table 1:** Experimentally obtained values for % extraction yield, total phenolic content, total flavonoid content, and % radical scavenging activity of hemp treatment groups

Hemp varitey	Extraction solvent	Extraction yield (%)	Total phenolic content (mg/100 g GAE)	Total flavonoid content (mg/100 g CE)	Radical scavenging activity (%)
Jinma 1	Ethanol	13.04 ± 0.43^Ba^	192.02 ± 1.19^Ba^	35.07 ± 3.72^Ca^	65.57 ± 0.24^Bb^
Cherry Wine	Ethanol	16.95 ± 0.64^ABa^	279.87 ± 1.68^Ba^	297.23 ± 9.16^Ba^	62.18 ± 0.10^Bb^
Queen Dream	Ethanol	24.08 ± 0.18^Aa^	319.34 ± 4.52^Aa^	538.26 ± 18.45^Aa^	65.77 ± 0.23^Bb^
Rogue	Ethanol	25.29 ± 0.70^ABa^	302.01 ± 1.67^Aa^	591.70 ± 15.69^Aa^	64.03 ± 0.26^Bb^
Jinma 1	Acetone	11.94 ± 0.63^Ba^	265.39 ± 5.59^Ba^	298.99 ± 0.58^Ca^	34.40 ± 0.26^Bc^
Cherry Wine	Acetone	21.03 ± 0.56^Aba^	212.92 ± 1.07^Ba^	296.79 ± 5.32^Ba^	13.09 ± 0.57^Bc^
Queen Dream	Acetone	23.14 ± 0.63^Aa^	296.51 ± 11.37^Aa^	522.32 ± 11.32^Aa^	35.51 ± 1.21^Bc^
Rogue	Acetone	21.77 ± 0.58^ABa^	372.85 ± 1.37^Aa^	453.64 ± 12.57^Aa^	46.41 ± 2.39^Bc^
Jinma 1	Deionized Water	4.79 ± 0.02^Bb^	131.59 ± 2.61^Bb^	100.02 ± 4.79^Cb^	-
Cherry Wine	Deionized Water	7.45 ± 0.31^ABb^	135.61 ± 1.16^Bb^	325.74 ± 6.74^Bb^	-
Queen Dream	Deionized Water	11.16 ± 0.29^Ab^	193.74 ± 8.32^Ab^	306.62 ± 5.39^Ab^	-
Rogue	Deionized Water	5.12 ± 0.65^ABb^	138.99 ± 1.01^Ab^	282.10 ± 4.12^Ab^	-
Ascorbic Acid	-	-	-	-	89.29 ± 0.45^Aa^

The results are presented in mean ± SEM values with the same lowercase letters indicating the same level of significance in columns between solvents (*p* ≤ 0.05; *n* = 3); mean values with the same uppercase letters indicate the same level of significance in column between varieties (*p* ≤ 0.05)

**Table 2:** Concentrations of different crude hemp extracts with different solvents

Hemp variety	Extraction solvent	Concentration mg/mL
Jinma 1	Ethanol	6.67
Cherry Wine	Ethanol	30.13
Queen Dream	Ethanol	33.33
Rogue	Ethanol	33.33
Jinma 1	Acetone	3.33
Cherry Wine	Acetone	13.33
Queen Dream	Acetone	16.67
Rogue	Acetone	23.33
Jinma 1	Deionized Water	3.33
Cherry Wine	Deionized Water	3.33
Queen Dream	Deionized Water	3.33
Rogue	Deionized Water	3.33

A significant interaction (*p* < 0.05) between the solvent type and the hemp varieties was noted to have influenced the EY. Therefore, the solvent-to-solvent interaction indicates the most effective extraction solvents for the varieties grown in northern Alabama. Ethanol and acetone solvents yielded higher results compared to water due to their lower polarity. This led to an increase in yield when high concentrations of phenolic acids were used. *Cannabis sativa* also contains significant components of terpenes, flavonoids, and cannabinoids as major secondary metabolite constituents. These components have lipophilicity and higher solubility in organic acids such as acetone and ethanol, thus resulting in a high yield (Do et al., 2014).

The highest yield was recorded in the ethanolic extracts for all treatments (13.04 ± 0.43% for J1E and 25.29 ± 0.70% for RE). Drinic et al. (2018) reported EY of 8.16 to 8.26% using 90% ethanol maceration of Hemp fluorescence, while 16.88 to 19.56% for water extracts. Ahmed et al. (2019) reported EY of 3% for acetone, 7% for ethanol, and 12% for water. This difference may have been due to biotic and abiotic factors, for example, solvent-to-solid ratio (5:25), extraction time (24 and 72 h), and hemp growing locations (Serbia and China) may have influenced the extraction yield (Pavlovic et al. 2018; Hanuš and Hod, 2020).

### Total flavonoid and phenolic acid contents

This study measured the total phenolic and flavonoid contents to evaluate the polyphenolic content extracted from different hemp varieties. Both contents quantify the amounts of phenolic compounds through a redox reaction. The total phenolic content is measured using the Folin-Ciocalteu reagent, which contains phosphomolybdic and phosphotungstic acids. Phenolic compounds consist of aromatic rings with hydroxyl groups, organic acids, and acylated sugars, all of which can reduce these two compounds, resulting in a color change to blue with a broad maximum absorption at 765 nm (Ahmed et al. 2019; Wanigasekera et al., 2019). Total flavonoid content was estimated based on the ability of flavonoid C-4 keto groups and C-3 or C-5 hydroxyl groups to form acid-stable complexes with Aluminum chloride (AlCl_3_) and can be detected using a colorimetric method at 510 nm (Wanigasekera et al., 2019).

TPC and TFC contents for hemp samples are shown in Table 1. TPC of hemp varieties ranged from 131.59 ± 2.61 mg/100 g dw GAE to 372.85 ± 1.37 mg/100 g dw GAE. TFC ranged from 35.70 ± 3.72 mg/100 g dw QE to 591.70 ± 15.69 mg/ 100 g dw QE. The highest TPC and TFCs were observed in the RAE and RE treatments. There was no significant difference between the acetone and ethanol solvents in the TPC and TFC treatments (*p* ≥ 0.05); the solvent enhanced the TPC concentration in the following order: water < acetone < = ethanol and variety J1 < CW < QD ≤ R. The solvent reduced the TFC content in the following order: ethanol ≥ acetone > water. TPC content variety decreased by variety in the following order: R ≥ QD > CW > J1.

Significant differences in TPC and TFC contents were observed between extraction solvents and hemp varieties. Extraction solvents of acetone and water resulted in significantly higher TPC and TFC, possibly due to the polarity of the solvents. Solvent polarity is determined by the dielectric constant, which measures a solvent’s capacity to insulate opposite charges from each other. Solvents with suitable polarity for the phytochemicals of interest can effectively extract them from the plant material matrix (Yahia et al. 2020; Daneshzadeh et al., 2019). As reported by Snyder (1978), the solvent polarity index for acetone and ethanol is 5.1 and 4.3, respectively, on a scale of 0–10 (with 0 representing the least polar (hexane), while to 10 extremely polar (water). The significant interaction found in both TFC and TPC for ethanol-acetone, Queen Dream and Rogue varieties shows that the extraction solvent and variety work in synergy to provide higher extraction of phenolic compounds due to the excellent miscibility of the solvent (Wakeel et al. 2019). This means that the polarity of acetone and ethanol falls within the range required to solubilize phenolic and flavonoid compounds. Differences in varieties may be explained by the various abiotic and biotic factors that influence the impact of chemotype on phenolic compounds. Thus, similar findings for TPC and TFC were reported in other Hemp and medicinal plant maceration (Drinic et al., 2018; Ahmed et al. 2019; Wakeel et al. 2019).

### Antioxidant activity

DPPH is an antioxidative assay based on the ability of a phytochemical group to stabilize the DPPH free radical. DPPH is a stable free radical because of the delocalization of an extra electron over the molecule, preventing DPPH from dimerizing. This allows the molecule to be scavenged (stabilized) by antioxidant phytochemicals such as flavonoids, phenolic acids, and cannabinoids through a hydrogen atom transfer mechanism. This process leads to a color change of a solution from a deep violet color that can absorb light at 517 nm (Do et al., 2014; Wanigasekera et al., 2019).

The results of the DPPH assay of the extracts are shown in Table 1. Hemp extracts radical scavenging activity ranged from 12.55 ± 0.39% (CWAE) to 65.68 ± 0.28 (J1E). All tested extracts of *C. sativa* showed a significantly lower (*p* ≤ 0.05) RSA when compared with the ascorbic acid. Although there were no significant differences between the varieties (*p* = 0.1621), there were significant differences between the solvents, and a significant interaction between the solvent and the variety (*p* ≤ 0.0001). Similar results were reported for macerated ethanolic and acetone extracts, with Naz (2016) reporting ethanolic and acetone extract RSA at 71.56 and 58.22%, and Ahmed et al. (2019) reporting 54.1 and 55.57% for ethanol and acetone, respectively.

The significant difference (*p* ≤ 0.0001) observed between the RSA’s of ethanol and acetone solvents, and the significant interaction between solvent and variety, may be attributed to the slight increase in polarity of ethanol as compared with acetone. This difference could have led to the solubilization of more phenolic and terpenophenolic compounds, as indicated by the presence shown in TPC and TFC evaluation, during the maceration process. These compounds may have had hydroxyl groups bonded to aromatic rings, enabling hydrogen atom transfer to occur, resulting in higher antioxidant activity in ethanolic extracts (Franco et al. 2008; Putri et al., 2019).

### Antimicrobial activity

**Fig. 1:**
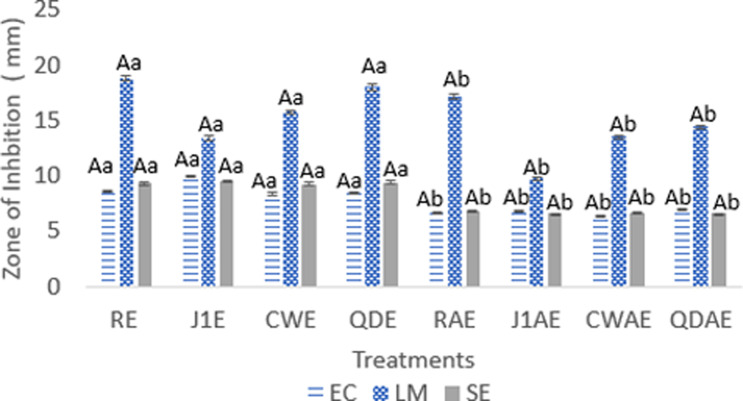
Bacterial growth inhibition zones were measured using the agar disc diffusion method after treatment with hemp extracts. The results are presented as mean ± SEM values (*n* = 3). Lowercase letters indicate the same level of significance in columns between solvents (*p* ≤ 0.05), while uppercase letters indicate the same level of significance between varieties (*p* ≤ 0.05). EC-*Escherichia coli O157:H7*, SE-*Salmonella enterica*, and LM-*Listeria monocytogenes*. Hemp Varieties: J1-Jinma 1, CW-Cherry Wine, QD-Queen Dream, R-Rogue. Solvents: E-Ethanol, AE-Acetone, and DIW-Deionized Distilled Water

**Fig. 2:**
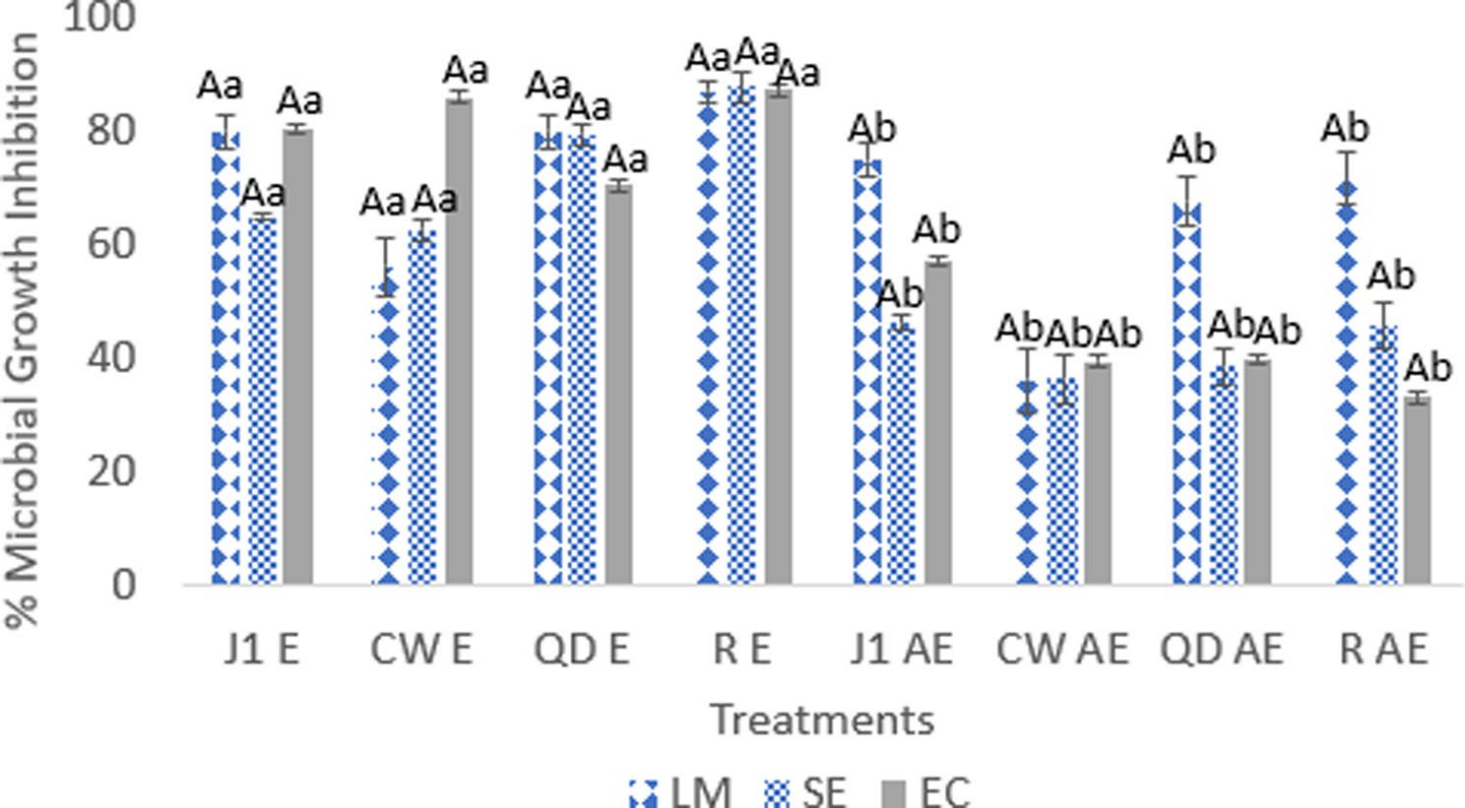
Bacterial growth inhibition with hemp extracts evaluated in a 96-well plate broth immersion. The results are presented as mean ± SEM values (*n* = 3). Lowercase letters indicate the same level of significance in columns between solvents (*p* ≤ 0.05), while uppercase letters indicate the same level of significance between varieties (*p* ≤ 0.05). EC-*Escherichia coli O157:H7*, SE-*Salmonella enterica*, and LM-*Listeria monocytogenes*. Hemp Varieties: J1-Jinma 1, CW-Cherry Wine, QD-Queen Dream, R-Rogue. Solvents: E-Ethanol, AE-Acetone, and DIW-Deionized Distilled Water

To survive, plants have developed natural defenses through the production of secondary metabolites to mitigate predation by herbivory and pathogenic microbes (viruses, bacteria, and fungi) as well as to protect against light radiation. These secondary metabolites have antimicrobial activity that can be extracted to combat these external threats, with bacteria being the primary focus of the present study (Yahia et al. 2020; Panphut et al. 2020). The antibacterial activity of plant extract depends mainly on its major phytochemical components, such as phenolics and terphenophenolics present in the plant, and its affinity for the extraction solvent (Daneshzadeh et al., 2019). Figures 1 and 2 show the results of hemp extract disc diffusion and 96-well plate growth inhibition against EC, SE, and LM. Hemp extracts zone of inhibitions were between 6.42 ± 0.02 (CWAE) to 9.99 ± 0.09 (J1E), 6.60 ± 0.04 (J1AE) to 9.57 ± 0.06 (J1E), and 9.74 ± 0.11 (J1AE) to 18.83 ± 0.26 (RE) for EC, SE, and LM respectively. Growth inhibition of hemp extracts against EC, SE, and LM ranged from 33.77 ± 3.998% (RAE) to 87.30 ± 2.46% (RE), 36.47 ± 1.71% (CWAE) to 87.81 ± 0.76% (RE), 36.20 ± 1.66% (CWAE) to 87.17% ± 0.52% (RE). Variety was not significant (*p* ≥ 0.05); however, solvent and the interaction between solvent and variety were significant (*p* ≤ 0.05).

There were significant differences in hemp treatments, with ethanolic and acetone extracts having significantly higher antimicrobial activity than water. Water extracts exhibited no inhibitory effects in any of the assays. This can be attributed to the polarity of the water, which solvates the phytochemical compounds in these hemp varieties. These compounds may have been less polar, preventing them from being solubilized in sufficient quantities to exhibit antimicrobial activity. In terms of percentage extraction yield, it was reported that water extracts had significantly lower extraction yields as compared with acetone and ethanol solvents (De Zoysa et al., 2019). Ethanolic extracts in both antibacterial assays displayed the highest inhibition in all extraction solvents (*p* ≤ 0.05) and were considered strong inhibitors (> 70%). This may be attributed in part to ethanol’s ability to extract more bioactive compounds due to its broad range in polarity. The type of solvent used in an antibacterial study has a significant impact on the extraction and efficiency of phytochemical. At present, the antibacterial mechanism of action of cannabinoids is still being determined. However, the antibacterial mode of action of other phytochemicals found in *Cannabis sativa* (400–500 phytochemical compounds, such as flavonoids and phenolic acids) have been proposed to work synergistically in what is called an entourage effect. This effect is explained by the increased activity of an active compound with an inactive one or 1 + 0 > 1 where phytochemical compounds work together to produce an antimicrobial effect greater than any compound acting alone (Hanuš and Hod, 2020). The proposed mechanism of action of *Cannabis sativa* phenolics is related to the alteration of membrane permeability due to reduced fluidity of the membrane and lipid segregation, resulting in reduced cell wall integrity and structure. This leads to the leakage of intracellular components (Schofs et al., 2021).

Disc diffusion antibacterial zone of inhibition activity has been classified into four main groups: 0- no inhibition, <8 mm weak inhibition, 8 mm < x < 10 mm moderate inhibition, and > 10 mm strong inhibition (Daneshzadeh et al., 2019). Disc diffusion results in this study showed that the ethanolic extracts were > 8 mm effective against SE and EC gram-negative bacteria and > 10 mm effective against LM gram-positive bacteria. These results are similar to those reported for other hemp and medicinal plant extracts (Abubakar et al. 2020; Yahia et al. 2020; Shah et al. 2019; Manandhar et al. 2019). Differences in the zone of inhibition of the hemp extracts between gram-negative and positive bacteria may reside in the bacterial cell wall morphological differences and provide different sensitivities to plant extracts (Schofs et al., 2021).

Gram-negative bacteria have structural lipopolysaccharide components on their outer phospholipid membrane, which makes their cell more resistant to antimicrobials (De Zoysa et al. 2019). On the other hand, Gram-positive bacteria have a hydrophilic thick peptidoglycan layer that is more permeable to plant phytochemicals. This is due in part to electrostatic interaction that allows phytochemicals to readily bind and diffuse into the inner cell membrane, leading to pore formation and a cascade of mechanisms. These mechanisms include membrane hyperpolarization, cytoplasmic coagulation, alteration of DNA, and leakage of cytoplasmic metabolites and ions, ultimately resulting in necrosis (Cushnie and Lamb 2005; Biharee et al. 2020).

### Pearson correlation and principal component analysis

**Fig. 3:**
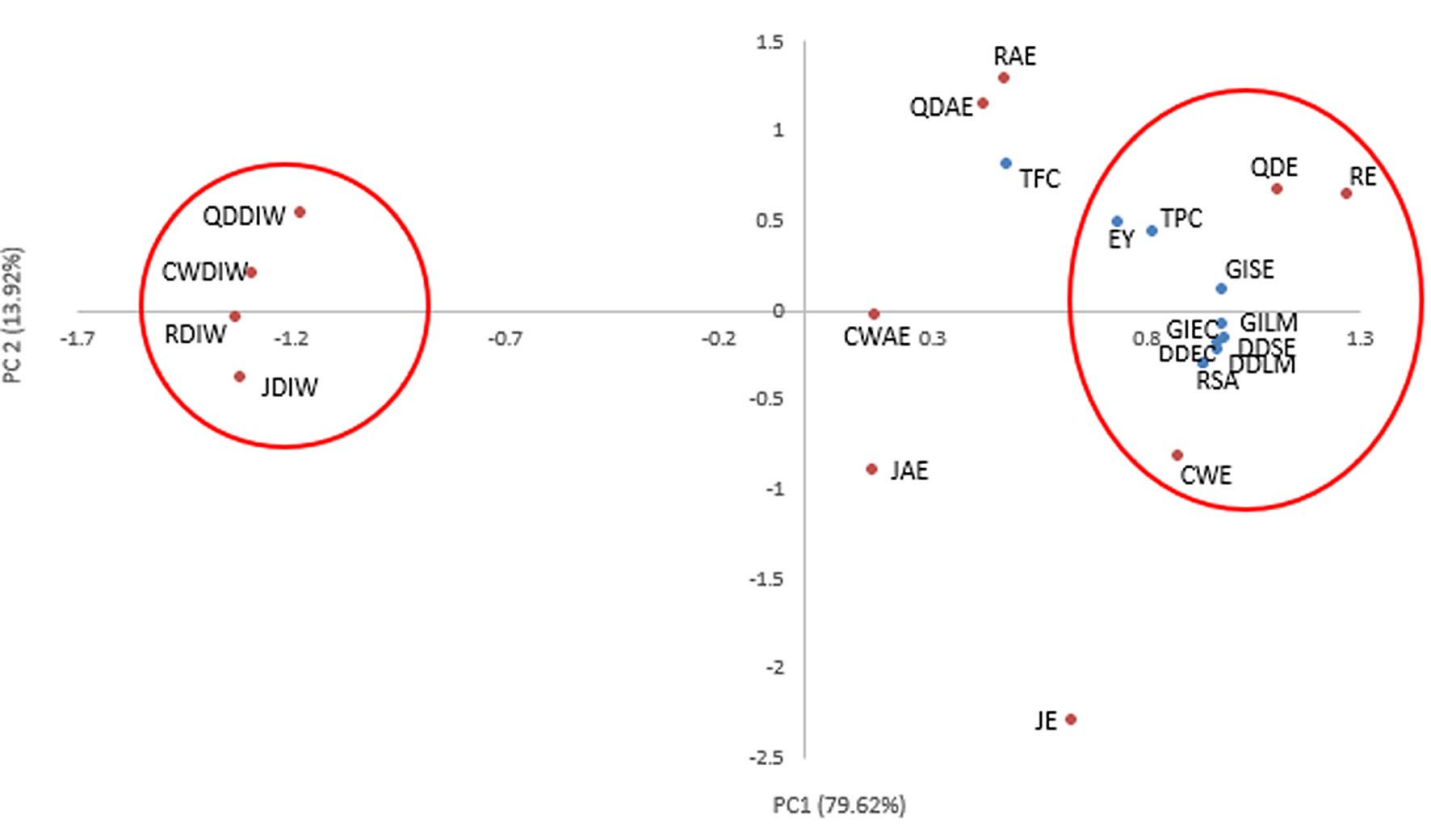
Principal component analysis of variables including phenolic content (TPC and TFC), extraction yield (EY), antioxidant activity (RSA), and antibacterial activities (DD-disc diffusion and GI-growth inhibition) of hemp extract treatments: J1-Jinma 1, CW-Cherry Wine, QD-Queen Dream, and R-Rogue; Solvents- E-Ethanol, AE-Acetone, and DIW-Deionized Water

The correlation between extraction yield, phenolic content (TFC and TPC), antioxidant activity (DPPH RSA), and antibacterial activity (disc diffusion (DD) and growth inhibition) was analyzed using a Pearson’s correlation test, and the results are presented in Table 3. In addition to the Pearson Correlation, a Principal Components Analysis was performed to better visualize the relationships among the evaluated variables of phenolic contents, extraction yield, and antioxidant and antibacterial activities in relation to hemp treatments (Fig. 3). A total of 93.54% of data variability can be explained by the first two factors (PC1 and PC2) in Fig. 3. As reported by Boateng and Yang (2021), a cumulative PC > 85% is sufficient to explain the variation across the dataset, indicating that the variables were closely related and share some characteristics. In the biplot in Fig. 3, the water extracts from all hemp varieties appeared on the negative side of PC1, while the acetone and ethanolic treatments were on the positive side of PC1, along with the evaluation variables (that is, phenolic contents, extraction yield, and bioactivities). Ethanolic solvent treatments clustered closer to the variables, suggesting that ethanolic treatment may be the optimal solvent for extraction. Regarding phenolic contents and antioxidant activity, there was a significant positive correlation between phenolic contents and radical scavenging activity (*p* ≤ 0.01). Antibacterial assays showed a significant positive correlation with each other and antioxidant activity (*p* ≤ 0.01). TPC was strongly correlated with all antibacterial assays (*r* ≥ 0.55).

**Table 3:** Correlation between extraction yield, antioxidant activity (radical scavenging activity), phenolic content (total flavonoid content and total phenolic content), and antibacterial activity (disc diffusion and growth inhibition) in hemp extracts using Pearson correlation coefficient (r)

	Extraction yield	Total flavonoid content	Total phenolic content	Radical scavenging activity	Disc diffusion against EC	Disc diffusion against SE	Disc diffusion against LM	Growth inhibition against EC	Growth inhibition against SE	Growth inhibition against LM
**Extraction Yield**	1.000	NS	NS	NS	-0.32374*	NS	NS	NS	NS	NS
**Total Flavonoid Content**	NS	1.000	0.64415**	0.42561**	NS	NS	0.48274**	NS	0.40612**	NS
**Total Phenolic Content**	NS	0.63559**	1.000	0.64415**	0.63108**	0.6492**	0.75406**	0.55387**	0.67426**	0.55387**
**Radical Scavenging Activity**	NS	0.42561*	0.64415**	1.000	0.87684**	0.90776**	0.8762**	0.93699**	0.91240**	0.93699**
**Disc diffusion v EC**	-0.32374*	NS	0.63108**	0.87684**	1.000	0.98573**	0.93806**	0.92876**	0.92088**	0.92876**
**Disc diffusion v SE**	NS	NS	0.6492**	0.90776**	0.9857**	1.000	0.95047**	0.93504**	0.93816**	0.93504**
**Disc diffusion against LM**	NS	0.48274**	0.75406**	0.8762**	0.93806**	0.95047**	1.000	0.84554**	0.90645**	0.84554**
**Growth Inhibition against EC**	NS	NS	0.55387**	0.93699**	0.92876**	0.93504**	0.84554**	1.000	0.92756**	1.0000**
**Growth Inhibition against SE**	NS	0.40612**	0.67426**	0.91240**	0.92088**	0.93816**	0.90645**	0.92756**	1.000	0.92756**
**Growth Inhibition against LM**	NS	NS	0.55387**	0.93699**	0.92876**	0.93504**	0.84554**	1.0000**	0.92756**	1.000

NS-no significant correlation, *significant with *p* ≤ 0.05, **significant correlation with *p* ≤ 0.01. EC-*Escherichia coli O157:H7*, SE-*Salmonella enterica*, and LM-*Listeria monocytogenes*

A significant positive correlation between phenolic content and radical scavenging activity was previously reported by Suleria et al. (2020). The study found a significant strong correlation between TPC and antioxidant activity (*r* = 0.932; *p* ≤ 0.01), and this is in line with the findings of the present study (*r* = 0.644; *p* ≤ 0.01). This suggests that phenolic compounds are major contributors to the antioxidant activity of hemp extracts. Positive significant correlations between the antibacterial and phenolic acid content were observed in our treatments (*r* ≥ 0.55). El-Chaghaby et al. (2014) found similar results when evaluating the antibacterial efficacy of *Annona squamosa* leaf extract against *E. coli* (EC) and *Staphylococcus aureus* (SA) and DPPH RSA. They reported significant positive correlations between RSA and growth inhibition of both SA and EC (*r* ≥ 0.85). Bendini et al. (2006) also reported a significant positive correlation between radical scavenging and growth inhibition activities. A significant positive correlation of bioactivity of hemp extracts may be attributed to the presence of phenolic and terpenophenolic compounds; these compounds have hydroxyl groups that can interact with the cell membrane of bacteria, leading to the degradation of the membrane composition and loss of cellular components. In addition, the hydroxyl groups can participate in electron transfer and donate hydrogen atoms, contributing to the stabilization of free radical species (Franco et al. 2008; Silva-Beltran et al., 2015).

## Conclusions

In various reports, hemp has been shown to contain phytochemical compounds (such as phenolics, flavonoids, and terpenophenolics) that effectively inhibit the growth of pathogenic bacteria and scavenge free radicals. Hemp has been used in traditional medicine as a therapeutic agent with antibacterial, anti-inflammatory, and chemopreventive properties that can cure many ailments. The ability of the hemp ethanolic extracts to scavenge the DPPH free radical indicates that they may have antioxidant properties. The inhibition of EC, SE, and LM in disc diffusion and growth inhibition assays by ethanolic hemp extracts suggests growth inhibitory effects of the extract, and pinpoints ethanol as the most effective extraction solvent for maceration extraction of northern Alabama varieties.

The obtained results support the idea that hemp grown in northern Alabama can be used as a plant-based natural preservative because of its antibacterial and antioxidant potential in food preservation. Future research is required to study quantitative antibacterial and antioxidant activities, mechanisms of antibacterial action, phytochemical profiles through analytical chromatography, and applications of hemp ethanol extract in nanotechnology.

## Data Availability

The datasets used and/or analyzed during the current study are available from the corresponding author on reasonable request.

## Abbreviations

EC: E. coli O157:H7
LM: Listeria monocytogenes
SE: Salmonella enterica
J1E: Jinma 1 Ethanol
CWE: Cherry Wine Ethanol
QDE: Queen Dream Ethanol
RE: Rogue Ethanol
J1AE: Jinma 1 Acetone
CWAE: Cherry Wine Acetone
QDAE: Queen Dream Acetone
RAE: Rogue Acetone
J1DIW: Jinma 1 Deionized Water
CWDIW: Cherry Wine Deionized Water
QDDIW: Queen Dream Deionized Water
RDIW: Rogue Deionized Water
AA: Ascorbic Acid
MHA: Mueller Hinton Agar
SPC: Standard Plate Count Agar
DD: Disc Diffusion
GI: Growth Inhibition
TPC: Total Phenolic Content
TFC: Total Flavonoid Content
DPPH: 2,2-Diphenyl-2-Picrylhydrazyl
ANOVA: Analysis of Variance
PCA: Principal Component Analysis
